# Colour Blindness

**Published:** 1893-02-11

**Authors:** 


### COLOUR BLINDNESS.

The report of the Commission on Colour Blindness is the subject of a somewhat discursive article in the *Edinburgh Review*. Too much attention cannot be attracted to a matter affecting the safety of all who travel by land or sea. Continued investigations have shown that the colour-blind may be divided into several distinct classes. Some few cannot distinguish blue from green; a still smaller number see only in monochrome (not, it is to be feared, for their sake, always in 'couleur de rose'). But the more common form is red-green blindness. A red-green blind man will regard a certain hue of green as identical with some hue of red; to another green and white will seem identical, while another will fail to see red at all of one particular hue. The late Emperor William was colour-blind, and was once known to compliment a lady in a bright scarlet gown on her thoughtfulness in remembering his favourite colour—well known to be cornflower blue. Now red, white, and green are used as safety and danger signals, by night and day, on land and sea. "No care is taken," says Mr. Bickerton, of Liverpool, before the Commission, "no care whatever, to prevent colour-blind boys from being brought up to the sea life. I examined all the youths on five training-ships, in all 1,056, and found among them a total of 34 colour blind, being specially trainep for a profession which they were physically and morally unfit to enter." Minute inquiry into the causes of several shipwrecks in late years have shown them to be due to the total inability of the helmsman to distinguish the signals. This deficiency in sight seems to be common in all nations. In Japan 68 out of 1,200 soldiers were found to be colour-blind. It can undoubtedly sometimes be traced to simple ignorance, and would decrease in extent if some system of colour training were adopted in all infant schools. The Commissioners issue a long list of recommendations, of which the most important are the formation of a schedule of employments from which colour blindness should exclude a candidate; a thorough examination of such candidates; and the adoption of a uniform tone and shade of colour and light for all signals on land and sea.

